# New Self-Organizing Optical Materials and Induced Polymorphic Phases of Their Mixtures Targeted for Energy Investigations

**DOI:** 10.3390/polym14030456

**Published:** 2022-01-23

**Authors:** Salhah H. Alrefaee, Hoda A. Ahmed, Mohd Taukeer Khan, Khulood A. Al-Ola, Hanaa AL-Refai, Mohamed A. El-Atawy

**Affiliations:** 1 Department of Chemistry, College of Science, Taibah University, Yanbu 30799, Saudi Arabia; srfaay@taibahu.edu.sa (S.H.A.); hhjuhani@taibahu.edu.sa (H.A.-R.); Maatawy@taibahu.edu.sa (M.A.E.-A.); 2 Department of Chemistry, Faculty of Science, Cairo University, Cairo 12613, Egypt; 3 Department of Physics, Faculty of Science, Islamic University of Madinah, Al-Madinah Al-Munawwarah 42351, Saudi Arabia; khanmtk@iu.edu.sa; 4 Chemistry Department, College of Sciences, Madina Monawara, Taibah University, Al-Madina 30002, Saudi Arabia; kabualola@taibahu.edu.sa; 5 Chemistry Department, Faculty of Science, Alexandria University, P.O. Box 426 Ibrahemia, Alexandria 21321, Egypt

**Keywords:** fluorinated liquid crystals, optical properties, thin film, geometrical structure, polymorphic phases, energy gap

## Abstract

Herein, a new homologues series of fluorinated liquid crystal compounds, **I*n***, 4-(((4-fluorophenyl)imino)methyl)-2-methoxyphenyl 4-alkoxybenzoate were synthesized and its mesomorphic properties were investigated both experimentally and theoretically. The synthesized compounds were characterized by elemental analyzer, NMR, and FT-IR spectroscopy to deduce the molecular structures. The differential scanning calorimetry was employed to examine mesophase transitions whereas the polarized optical microscopy was used to identify the mesophases. The obtained results revealed that the purely nematic phase observed in all terminal side chains. All homologues showed to possess monotropic nematic mesophases except the derivative **I*8*** exhibits enantiotropic property. The optimized geometrical structures of the present designed groups have been derived theoretically. The experimental data was explained using density functional theory computations. The estimated values of dipole moment, polarizability, thermal energy, and molecule electrostatic potential demonstrated that the mesophase stability and type could be illustrated. Binary phase diagram was constructed and addressed in terms of the mesomorphic temperature range and obtained polymorphic phases. It was found that incorporation of the terminal F-atom and lateral CH_3_O group influence both conformation and steric effect in pure and mixed states. The absorption and fluorescence emission spectra of fabricated films were recorded to elucidate the impact of terminal side chain on photophysical properties of synthesized liquid crystal. It was noted that the increase of terminal side chain length lead to reduction of optical band gap, whereas charge carrier lifetime increases.

## 1. Introduction

Recently, the addition of a lateral fluorine atom has shown a significant impact on the optical and mesomeric properties of nematogenic compounds. The position and spatial orientation of fluorine atom further influence properties of nematogenic materials. The physical characteristics of the resulting liquid crystalline material, such as melting and phase transition temperatures, mesomorphic morphology, dipole moment, and dielectric anisotropy, were significantly influenced by the combination of the lateral fluorine atom’s small size and high polarity [1,2,3,4,5,6,7,8,9,10,11].

The mesomeric characteristics of Schiff bases/ester complexes are significantly affected by the lateral or terminal polar group [12], particularly the fluorine atom. Owing to their photoactivity under UV irradiation, azo and esters are also ideal linking groups for designing new mesomorphic structures [13,14,15,16,17,18,19]. Intermolecular separation is generally increased by lateral substitution, which extends the core moiety and decreases lateral molecular contacts [20,21,22,23,24].

Grey [25] discovered that increasing the molecule’s width lowers the stability of both the smectic and nematic phases. The fluorine atom’s small size allows the observation of liquid crystalline mesophases without causing sterical disruption. Additionally, the high polarity of fluorine atom influences the optical morphology, melting temperature, transition events, dielectric anisotropy, and other physical features. Furthermore, depending on the position and orientation, the polar fluorine atom also impacts the polarisability and dipole moment of the entire molecule structure. The mesomorphic features of the resulting molecule clearly reflect this. A small variation in molecular structure lead to changes in the polarity and direction of dipole moments, consequently, improvement of optical characteristics and observance of new mesophase behavior. The molecules, on the other hand, tend to orientate in a parallel arrangement as the length of the terminal substituent grows [26]. Furthermore, the twist-bend nematic and heliconical phases are influenced by the length of the terminal chains [27,28,29,30].

Remarkably, the computational assumption for new designing materials [31,32,33,34] shows a degree of attractiveness. To provide a wide range of optical properties, stimulated information about the molecule orbital energies and molecular geometries of liquid crystalline materials is required. Density functional theory (DFT) has recently emerged as a viable technique owing to its superior performance and reproducible computational results. Further, the knowledge of optical and photophysical properties of liquid crystal material is important to find its application in optoelectronic devices. The absorption spectroscopy is the primary tool to check whether a material is suitable for solar cells active layer or not by evaluating its optical band gap and absorption coefficient [35]. The steady state and time resolved fluorescence spectroscopy is used to illustrate the charge carrier dynamics in a semiconducting material under illumination [36].

**Figure 1:**
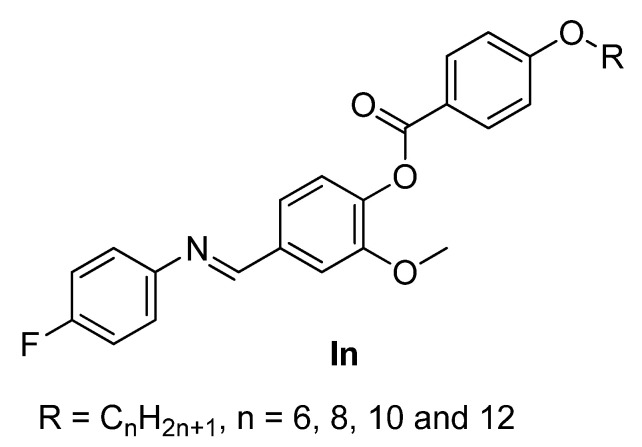
Structure of 4-(((4-fluorophenyl)imino)methyl)-2-methoxyphenyl 4-alkoxybenzoate **I*n***.

The goal of the present work is to synthesize new fluorinated liquid crystals, 4-(((4-fluorophenyl)imino)methyl)-2-methoxyphenyl 4-alkoxybenzoate, (**I*n***), possessing two un-symmetrical terminal substituents (Figure 1). The first terminal is an alkoxy chains with 6, 8, 10, and 12 16 carbons connected to the phenyl benzoate moiety. The second wing is the F-atom attached to the phenylimine mesogen. Experimentally, we investigate the mesomorphic and photophysical properties of synthesized materials and theoretically, predicted parameters by employing DFT calculation and investigated the impact of the linking group, alkoxy chain length as well as the position of lateral and terminal substituents. The optical properties of synthesized materials was investigated by recording absorption spectra, whereas steady state and time resolved fluorescence spectroscopy were employed to examine the impact of alkoxy side chain length on photophysical properties of liquid crystal.

## 2. Experimental

### 2.1. Synthesis

**Scheme 1:**
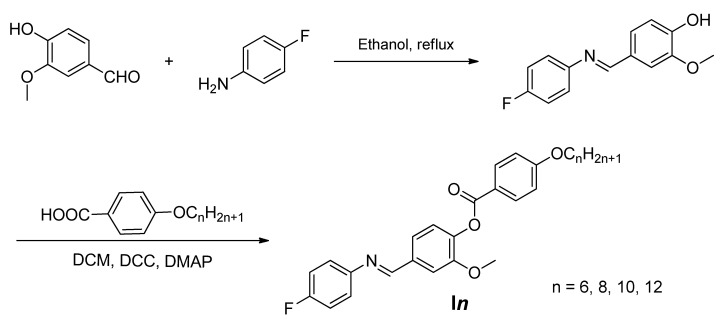
Synthesis of 4-(((4-fluorophenyl)imino)methyl)-2-methoxyphenyl 4-alkoxybenzoate, **I*n***.

The liquid crystals **I*n*** were synthesized according to Scheme 1.

#### 2.1.1. Synthesis of 4-(((4-Fluorophenyl)imino)methyl)-2-methoxyphenol

The methods of preparation are given in Supplementary Data [37,38].

#### 2.1.2. Synthesis 4-(((4-Fluorophenyl)imino)methyl)-2-methoxyphenyl 4-alkoxybenzoate, **I*n***

Preparation details are depicted in Supplementary Data.

4-(((4-Fluorophenyl)imino)methyl)-2-methoxyphenyl 4-(hexyloxy)benzoate **I*6***

Yield: 93.0%; mp 116.0 °C, FTIR (ύ, cm^−1^): 2912 2850 (CH_2_ stretching), 1732 (C=O), 1590 (C=N), 1612 (C=C). ^1^H NMR (500 MHz, DMSO-*d6*) *δ* ppm: δ 8.61 (s, 1H, CH=N), 8.03 (d, *J* = 7.3 Hz, 2H, Ar-H), 7.69 (s, 1H, Ar-H), 7.52 (d, *J* = 7.7 Hz, 1H, Ar-H), 7.37–7.31 (m, 3H, Ar-H), 7.26–7.17 (m, 2H, Ar-H), 7.08 (d, *J* = 7.5 Hz, 2H, Ar-H), 4.04 (s, 2H, OCH_2_), 3.80 (s, 3H, OCH_3_), 1.70 (m, 2H, CH_2_), 1.39 (m, 2H, CH_2_), 1.27 (m, 4H, 2CH_2_), 0.84 (m, 3H, CH_3_). ^13^C NMR (126 MHz, DMSO-D6) δ 163.86, 163.67, 160.40, 151.54, 147.58, 142.47, 135.31, 132.71, 123.91, 123.42, 122.75, 120.86, 116.48, 115.27, 111.77, 68.56 (OCH_2_), 56.47 (OCH_3_), 31.38 (CH_2_), 28.79 (CH_2_), 25.32 (CH_2_), 22.31 (CH_2_), 14.13 (CH_3_). Elemental analyses: Found (Calc.): C, 69.09 (68.96); H, 5.94 (6.11); N, 3.93 (3.95); F, 5.33 (5.49).

4-(((4-Fluorophenyl)imino)methyl)-2-methoxyphenyl 4-(octyloxy)benzoate **I*8***

Yield: 91.5.%; mp 97.0 °C, FTIR (ύ, cm^−1^): 2920 2855 (CH_2_ stretching), 1728 (C=O), 1592 (C=N), 1610 (C=C). ^1^H NMR (500 MHz, DMSO-d6) δ 8.60 (s, 1H, CH=N), 8.02 (d, *J* = 8.4 Hz, 2H, Ar-H), 7.69 (s, 1H, Ar-H), 7.52 (d, *J* = 8.0 Hz, 1H, Ar-H), 7.33 (m, 3H, Ar-H), 7.22 (t, *J* = 8.5 Hz, 2H, Ar-H), 7.06 (d, *J* = 8.4 Hz, 2H, Ar-H), 4.04 (t, 2H, OCH_2_), 3.80 (s, 3H, OCH_3_), 1.71 (m, 2H, CH_2_), 1.45–1.14 (m, 10H, 5 CH_2_), 0.81 (t, 3H, CH_3_). ^13^C NMR (126 MHz, DMSO-D6) δ 164.00, 163.84, 160.48, 152.00, 148.16, 142.74, 135.44, 132.62, 124.04, 123.39, 123.32, 122.83, 120.92, 116.49, 116.31, 115.34, 111.80, 67.95 (OCH_2_), 55.98 (OCH_3_), 31.65 (CH_2_), 29.23 (CH_2_), 29.16 (CH_2_), 29.02 (CH_2_), 25.85 (CH_2_), 22.96 (CH_2_), 14.35 (CH_3_). Elemental analyses: Found (Calc.): C, 72.93 (73.06); H, 6.75 (6.71); N, 2.93 (2.95); F, 3.98 (3.79).

4-(((4-Fluorophenyl)imino)methyl)-2-methoxyphenyl 4-(decyloxy)benzoate **I*10***

Yield: 92.1.%; mp 92.0 °C, FTIR (ύ, cm^−1^): 2918 28545 (CH_2_ stretching), 1725 (C=O), 1590 (C=N), 1613 (C=C). Elemental analyses: Found (Calc.): C, 73.64 (73.42); H, 7.18 (6.96); N, 2.77 (2.75); F, 3.76 (3.78).

4-(((4-Fluorophenyl)imino)methyl)-2-methoxyphenyl 4-(dodecyloxy)benzoate **I*12***

Yield: 93.4.%; mp 78.0 °C, FTIR (ύ, cm^−1^): 2925 2850 (CH_2_ stretching), 1730 (C=O), 1595 (C=N), 1615 (C=C). Elemental analyses: Found (Calc.): C, 74.27 (74.16); H, 7.55 (7.72); N, 2.62 (2.85); F, 3.56 (3.71). 

**Scheme 2:**
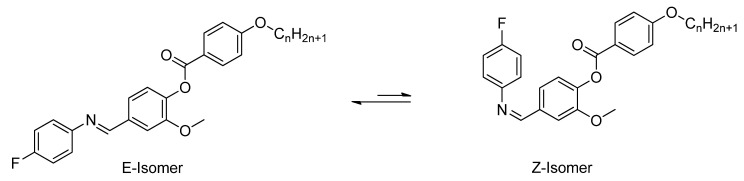
E- and Z- geometrical isomers of **I*n***.

The isomerization of the present laterally substituted Schiff bases (**I*n***) has been investigated. The presence of only one isomer (E-isomer) has been confirmed by NMR spectra. No duplicate peaks neither for azomethine protons nor for aromatic protons have been observed. This is owing to the steric hindrance between the bulky two phenyl groups which destabilizes the Z-isomer relative to the E-isomer (Scheme 2).

### 2.2. Characterization

Details are given in Supplementary Data.

### 2.3. Computational Method

DFT calculations for the investigated substances were performed using Gaussian 09 software [39]. The computations were performed using the DFT/B3LYP methods with the 6–31G (d,p) basis set. Without imposing any molecular symmetry limitations, the geometries were optimized by minimizing the energies with respect to all geometrical parameters. Gauss View [40] was used to design the structures of the optimized geometries. In addition, the same level of theory was used to calculate frequencies. In the geometry optimization method, the frequency calculations revealed that all structures were stationary points with no imaginary frequencies.

## 3. Results and Discussion

### 3.1. Liquid Crystalline Properties

**Figure 2:**
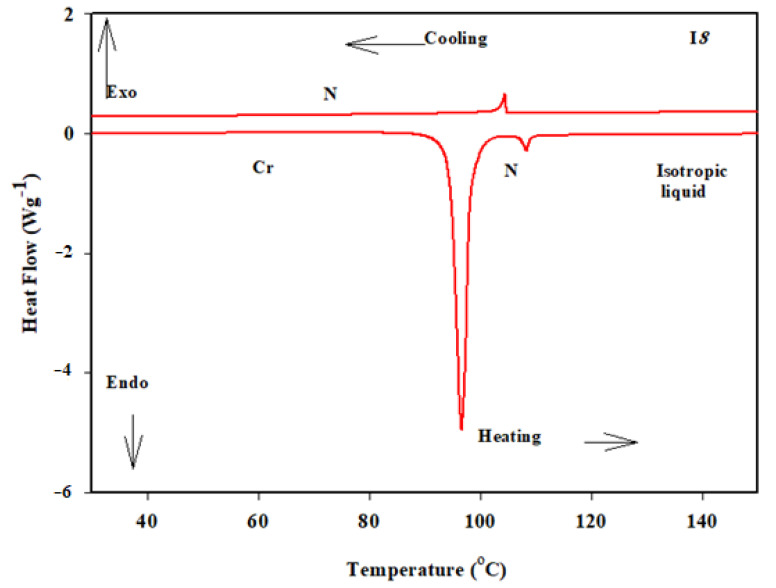
DSC thermograms recorded from the second heating and cooling scan with heating rate 10 °C/min of compound **I*8***.

**Figure 3:**
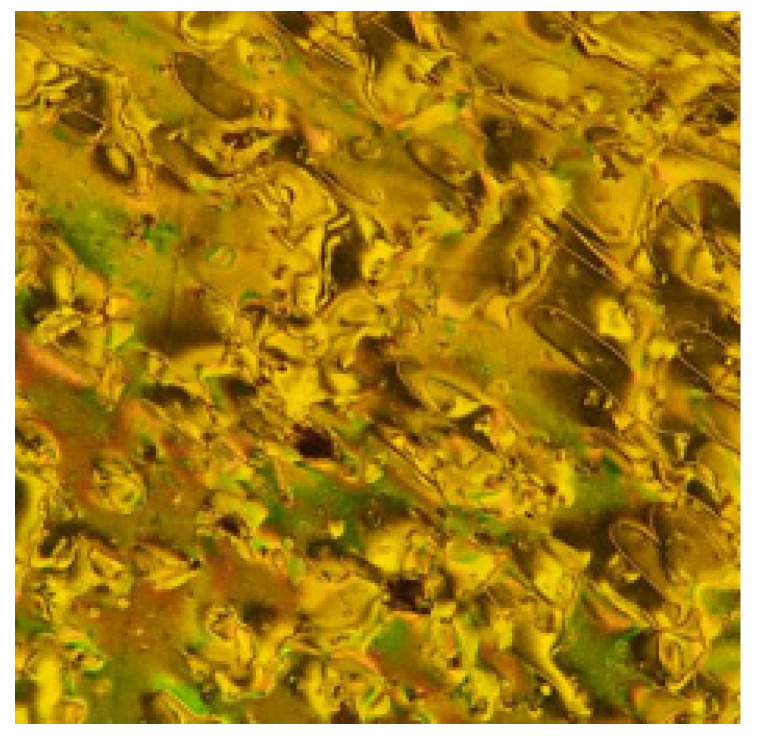
Nematic phase texture (529 × 609 px) observed under POM for compound **I*8*** at 100 °C.

**Figure 4:**
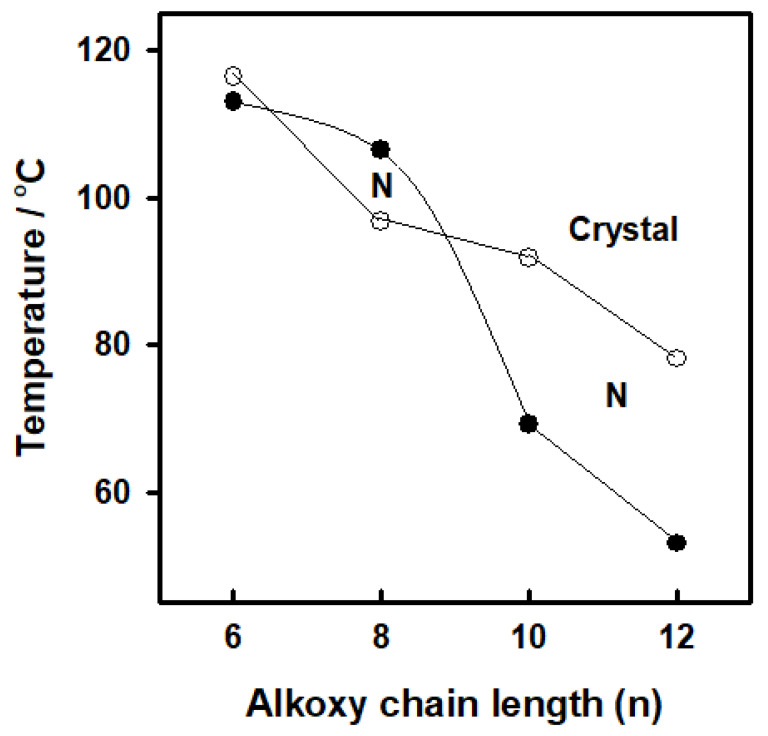
Effect of flexible alkoxy chain length (n) on the phase behavior of the homologous series **I*n*** upon cooling; (open circle) crystal phase and (closed circle) nematic phase.

The mesomorphic properties of the present synthesized series (**I*n***) were investigated. The results of the transition temperatures and enthalpies, as determined from the DSC investigations are collected in Table 1. DSC data were estimated from the second heating/cooling cycles to study the stability of the synthesized compounds. The second heating scan was used to record all of the thermal properties of these substances. DSC thermogram of the synthesized homologue I*8* is presented in Figure 2. On heating, the homologue showed two endothermic peaks assigned to the crystal-to-mesophase and mesophase-to-isotropic transitions while on cooling, a reversed exothermic peak was observed corresponds to isotropic to mesophases transition as presented in Figure 2 and the mssophase still present until room temperature. The POM textures was also confirmed by the DSC data. POM showed schlieren textures of the nematic (N) phase (Figure 3). All homologues exhibits the monotropic monomorphic characteristics except the derivative I*8* shows enantiotropic property. Figure 4 shows a graphical illustration of DSC temperature of transitions to evaluate the effect of the terminal alkoxy chains (n) on the mesomorphic behavior of the formed derivatives [41,42]. All examined compounds of the present series (**I*n***) are nematogenic, as shown in Table 1 and Figure 4. In general, the type of linking spacers and the length of terminal chains as well as the size of attached substituents determine the mesomorphic behavior for any designed liquid crystalline molecular architecture [26,38]. As shown from Table 1 and Figure 4, the melting transitions temperature follow a random pattern. The homologue **I*12*** possesses the lowest melting point (78.1 °C) whereas the homologue **I*6*** has the highest melting transition temperature (116.3 °C). All compounds of the prepared group are nematogenic and display purely N mesophases and their N phase stability decreases with the terminal alkoxy chain, *n*; these results are in consistent with previous documents [25,26,27,28,29,30,31,32,33,34,35,36,43,44,45].

In general, the mesogenic core of the molecule’s polarity and/or polarisability plays the most important roles in determining mesophase behavior. In comparison of present derivatives, homologue **I*6*** has the highest nematic stability (112.9 °C), whereas homologue **I*12*** has the lowest N phase stability (53.0 °C). Wide N temperature ranges are observed for all homologues upon cooling. From the present results, it could be concluded that, as the molecular anisotropy increases, as a result of the change in the mesogenic core of the molecule, the temperature range of the produced mesophases is increased.

On the other hand, the geometrical parameters such as dipole moment, polarizability, and molecular shape of the prepared homologues **I*n*** are highly affected on the association of molecules that leads to enhance the formation of wide N mesophase temperature range on cooling.

The normalized entropy changes, Δ*S*/R, upon cooling of the prepared compounds (**I*n***) are collected in Table 1. Independent of the alkoxy chains length (n), the entropy of N transition is of small magnitude. The lower value of entropy changes for N transitions of homologous series **I*n*** can be explained by the lower degree of linear alignments of the molecules at high temperatures. Furthermore, the terminal chains play a significant part in the molecule’s multi-conformational modifications [46].

### 3.2. Geometrical Structures

Because fluorine atom is one of smallest atoms, lateral fluorine inside the mesogenic part of the molecule will have a slight steric effect. Furthermore, the fluoro substituent has the maximum electronegativity and is very polar (3.98). It also has a low polarizability (5.57 × 10^−25^ cm^−1^), which means that intermolecular dispersion interactions are minimal. The effect of fluoro substitution in a laterally substituted mesogen, on the other hand, is largely reliant on its location.

**Figure 5:**
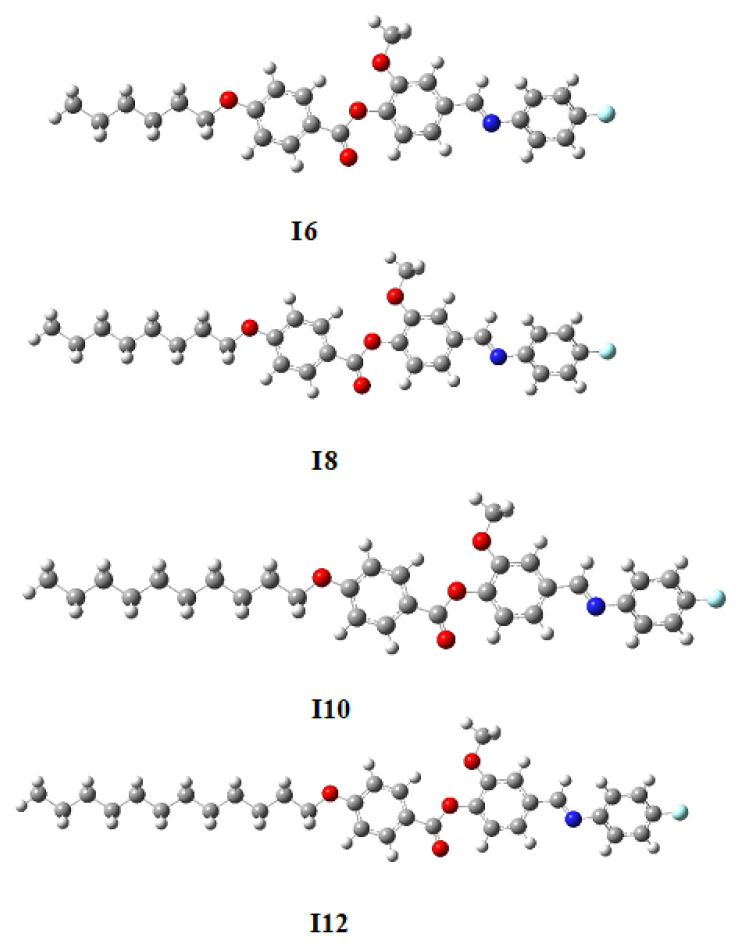
Optimized structures of molecules for the present series **I*n***, calculated at B3LYP/6-311G ** level.

The optimized geometrical shape of designed fluorinated derivatives **I*6*, I*8*, I*10*** and **I*12*** are shown in Figure 5. The computational calculations were performed by DFT/B3LYP method using 6–31G (d,p) basis set and GAUSSIAN 09W. The calculated data predict that the all compounds are linear. The synthesized homologues (**I*n***) should be in planar conformation because they are mesomorphic. The frequency calculation confirmed that the optimized structure of each member of the current series is stable, as no imaginary frequency was predicted for any of the members (Figure 5).

It is widely known that the polarizability, dipole moment and total thermal energy of liquid crystalline materials have a significant impact on the type and mesophase stability [13]. Furthermore, these parameters have an impact on some properties that are significant for nonlinear optical (NLO) liquid crystal applications such as optical interconnections, telecommunications, and signal processing [47,48]. The computed energies of the highest occupied molecular orbital (HOMO) and the lowest unoccupied molecular orbital (LUMO), as well as the energy difference between them, using the same approach and the 6–31G (d,p) basis set for all compounds have been calculated. The predicted geometrical and thermal parameters values are collected in Table 2 and Table 3. Results revealed that the competition between the intermolecular terminal and lateral molecular interactions impacts the mesomorphic characteristics. This competition leads to the predomination of one of them according to the geometrical structure optimization. The linear-shape of the investigated homologues (**I*n***) enhances the terminal interactions over the lateral ones. This consequently resulted in the production of the N mesophase, covering all of the prepared fluorinated derivatives (**I*n***).

**Figure 6:**
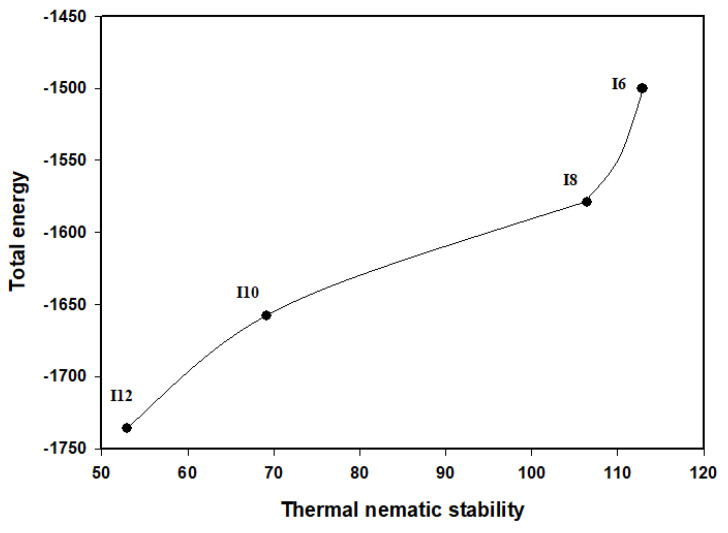
The total energies of the present series **I***n* relation with their nematic phase stability.

It was found that, linear dependency of the polarizability and dipole moment of the whole molecule as the terminal alkoxy chain length (n) increases (Table 3). Another linear dependency of the estimated total thermal energy as the stability of the N phase increases (Figure 6).

#### 3.2.1. Frontier Molecular Orbitals (FMOs)

**Figure 7:**
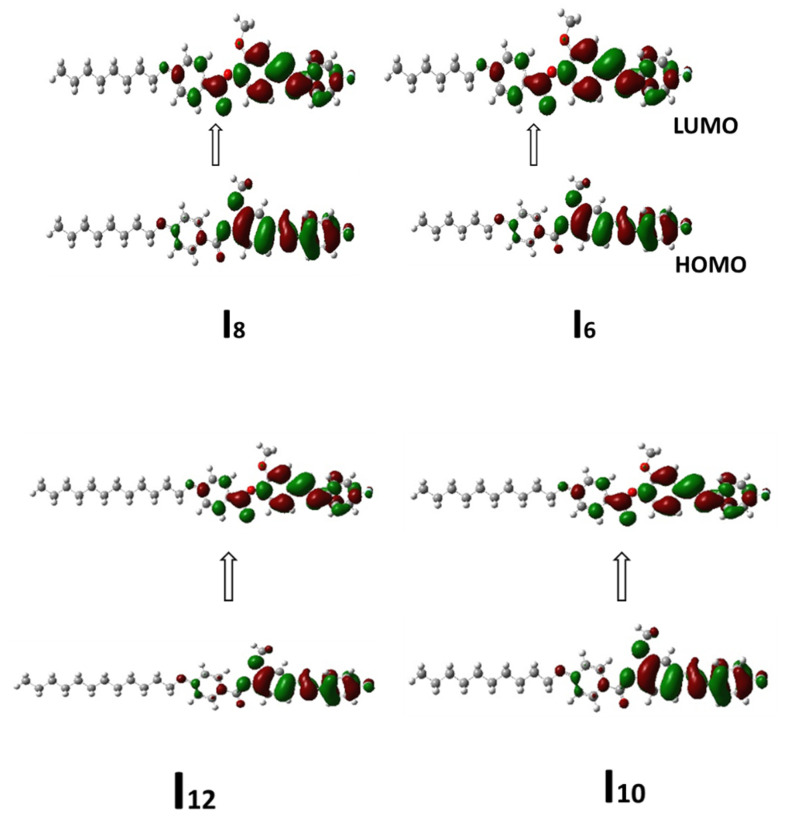
Estimated geometry for FMOs of the investigated series, **I*n***.

Figure 7 depicts the frontier molecular orbitals HOMO (highest occupied) and LUMO (lowest unoccupied) diagrams for the current fluorinated derivatives designs **I*n***. Table 3 lists the calculated energies and energy gap (∆E) values. The energy gap predicts the ability of electron transfer from HOMO to LUMO during the electronic excitation mechanism and is inversely connected with reactivity [49]. The electron densities of the sites involved in the production of HOMOs and LUMOs are localized on the fluoro substituent and azomethine moiety as well as lateral methoxy group, according to the expected values. Furthermore, the two terminals of molecules have a minor effect on the placement of the FMOs’ electron distributions. In addition, the energy gap of FMOs is not affected by the length of the alkoxy chain (n).

#### 3.2.2. Molecular Electrostatic Potential (MEP)

**Figure 8:**
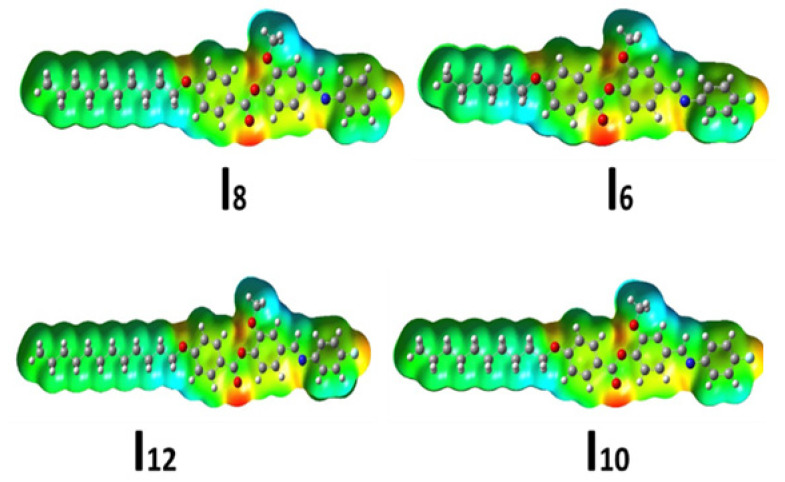
MEP mapped on the electron density surface for of homologues series **I*n***.

The distribution of electron density at atomic locations of LC materials affects molecule polarizability, electronic structure, dipole moment, and many other properties [36]. In addition, the molecular electrostatic potential (MEP) is a significant element for studying the electron density distribution in molecules [47,48,49] Furthermore, MEP is one of the most effective methods for determining whether or not a molecule has intermolecular and/or intramolecular interactions. The B3LYP/6311G(d,p) method was used to compute the MEP of the examined compounds (**I*n***, Figure 8). The position of the polar attached terminal F-group as well as the connecting azomethine and lateral methoxy groups of the examined compounds had slight effect on the localization of the iso-electronic density of the electron rich and electron deficient areas. The ester group of the benzoate component, on the other hand has the most electrons. While, the terminal alkoxy chain shows the least negatively charged atomic sites.

### 3.3. Binary Phase Diagram

**Figure 9:**
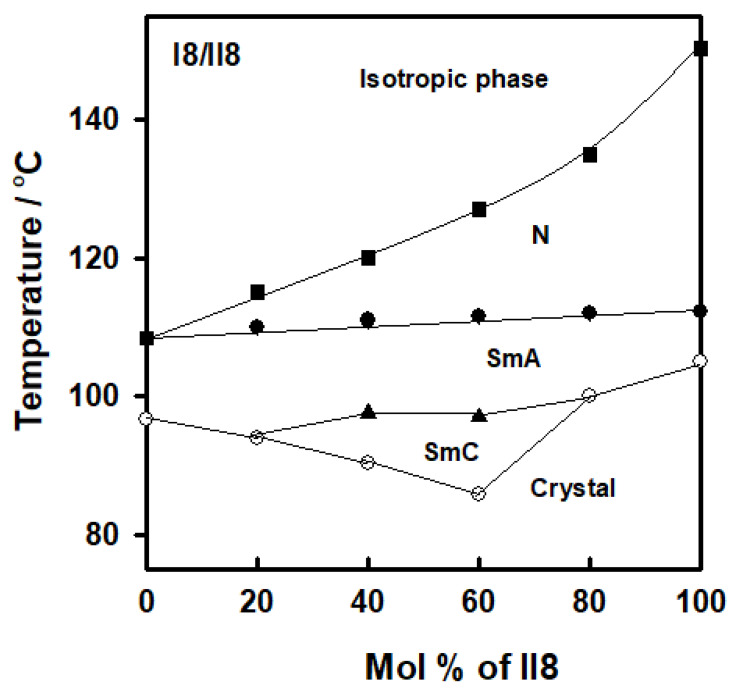
Binary phase diagram of **I*8***/**II*8*** system.

Figure 9 shows an example of a binary phase diagram derived by DSC investigations of two components bearing same alkoxy chain lengths, present derivative **I***8* and laterally neat, **II*8***. The binary mixtures was produced from the enantiotropic terminal chain homologues (**I*8*/II*8***). Laterally neat derivative **II*8*** exhibit the SmA and N mesophases enantiotropically. As seen in this figure, the binary phase diagram exhibits a negative depression of the N phase from the normal behavior. The little disruption of the N phase can be attributable to the great difference between the geometry the two components of the mixture that disturbs to some extent the linear arrangement of the molecules. Moreover, induced polymorphic phases have been produced at compositions 40 and 60 mol % of **II*8***. Figure 9 also demonstrates that the solid mixture with eutectic composition of 60.0 mol % of **I*16*** has a eutectic melting point of 85.7 °C and mesomorphic temperature range of 41.3 °C. It may be deduced that incorporation of the terminal F-atom and lateral methoxy group influence both conformation and steric effect in pure and mixed states that lead to formation of induced polymorphic phases.

### 3.4. UV-Vis Absorption Spectra

**Figure 10:**
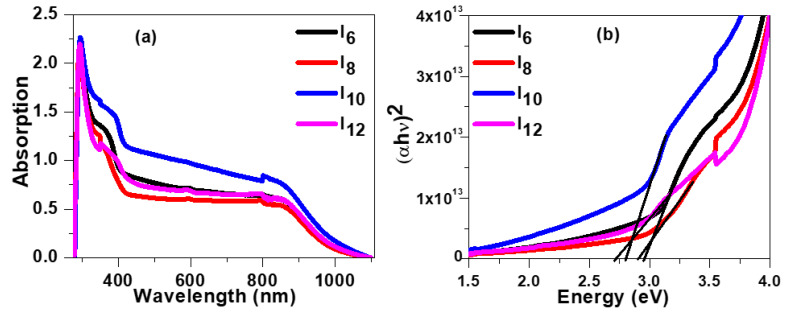
(**a**) Absorbance spectra of all samples with varying alkoxy side chain length. (**b**) Tauc plot demonstrating the evaluation of band gap.

The UV-Visible absorption spectra of prepared samples on glass slides are illustrated in Figure 10a. The spectra exhibit two regions: high energy absorption from 280 nm to 430 nm and low energy absorption from 430 nm to 950 nm. The peak absorption in higher energy region for all samples was observed at ~293 nm which correspond to $\pi -\pi^{*}$ transition in aromatic ring. The absorption in this region also exhibits a shoulder ~361 nm corresponds to absorption by side alkyl chain. As the length of side chain increases shoulder becomes more prominent and its position red shifted to 365 nm, 383 nm and 392 nm, for samples **I*8***, **I*10*** and **I*12***, respectively. The direct energy band gaps of fabricated samples were evaluated from Tauc plot shown in Figure 10b [50] using relation: ${\left(\alpha h\upsilon \right)}^{2}=A\left(E-E_{g}\right)$, where absorption coefficient $\alpha =\frac{Abs.}{Thickness}$ and *A* is an empirical constant. The bandgap of sample with smallest alkyl side chain was noted to be 2.97 eV and found to be decreases with increase of side chain length. The evaluated bandgap for samples **I*8***, **I*10*** and **I*12***, are 2.93 eV, 2.79 eV and 2.70 eV, respectively.

### 3.5. Fluorescence Spectra

**Figure 11:**
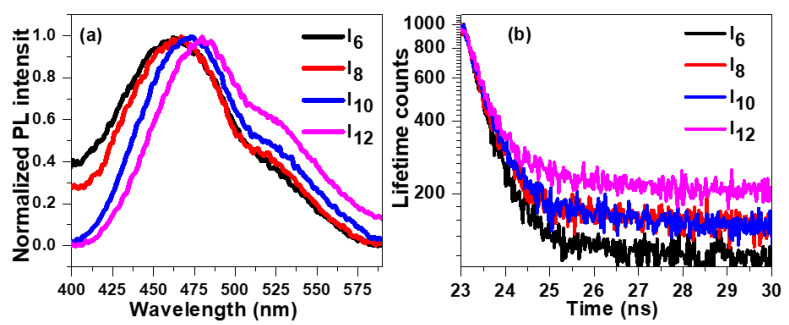
(**a**) Steady state and (**b**) time resolved fluorescence spectra of synthesized liquid crystal thin films.

To illuminate the influence of alkoxy side chain length on photophysical properties of prepared sample, we recorded steady-state photoluminescence (PL) spectra by exciting the sample via 320 nm laser diode. The normalized PL spectra of fabricated samples are displayed in Figure 11a which show a broad emission in the range 400–575 nm. The peak emission of **I*6*** sample was observed at 462 nm and noted to be red shifted with increase of flexible alkoxy side chain length. The peak emission of **I*8***, **I*10*** and **I*12*** samples were noted at 467 nm, 473 nm and 480 nm, respectively. The fluorescence decay spectra of these samples are shown in Figure 11b which were recorded at 465 nm. It can be noted from the Figure that as the alkyl side change length increases the fluorescence decay becomes slower. The excited state charge carrier lifetime in these samples were evaluated by fitting the decay spectra with single exponential decay: I(t)=A+Be−tτ, where τ is lifetime. The charge carrier lifetimes in **I*6*** sample was evaluated to be 107 ns. It can be noted from Table 4 that with increase of alkyl side chain length the lifetime increases attributed to the stearic hindrance of alkyl side chain. The lifetime **I*8***, **I*10*** and **I*12*** samples were calculated to 133 ns, 150 ns and 178 ns, respectively.

## 4. Conclusions

New nematiogenic fluorinated liquid crystal homologues series were prepared and examined for their mesophase and photophysical behavior for energy investigations. All materials are exhibit purely nematogenic mesophase monotropically except the derivative I8 possess enantiotropic nematic phase. Theoretical calculations revealed that, the linear-geometry of the investigated compounds enhances the terminal interactions and resulted in the production of the N mesophase covering all the terminal chains. The optical band gap of liquid crystal material was found to be decreases from 2.97 eV for **I*6*** to 2.70 eV **I*12*** attributed to improved crystallinity with increase of alkoxy side chain. The red shift in steady state fluorescence spectra and increase of charge carrier lifetime further confirm improvement of crystallinity of liquid crystal material with increase of terminal side chain length.

## Figures and Tables

**Table 1:** Phase transition temperatures (***T***, °C) upon heating and cooling cycles, enthalpy of transitions (**∆*H***, kJ/mol), and normalized transition entropy upon cooling (**∆*S***/R) of present series **I*n***.

Cycle	*Upon Heating*			*Upon Cooling*		∆*S*/R
**Comp.**	***T*** **_Cr-I_**	***T*** **_Cr-N_**	***T*** **_N-I_**	***T*** **_I-N_**	***T*** **_N-Cr_**	
**I*6***	116.3 (42.22)	-	-	112.9 (1.16)	-	0.36
**I*8***		96.7 (45.64)	108.4 (1.85)	106.5 (1.13)	*-*	0.36
**I*10***	91.7 (36.14)	-	-	69.2 (1.18)	21.7 (19.35)	0.41
**I*12***	78.1 (37.00)	-	-	53.0 (1.13)	32.33 (18.72)	0.42

Abbreviations: Cr-I = solid-isotropic liquid; Cr-N = solid-nematic; N-I = nematic-isotropic liquid; I-N = isotropic liquid-nematic; N-Cr = nematic-solid.

**Table 2:** DFT predicted thermal parameters for series **I*n***.

Compound	ZPE (Kcal/Mol)	Thermal Energy (Kcal/Mol)	Enthalpy (Kcal/Mol)	Gibbs Free Energy (Kcal/Mol)	Entropy (Cal mol.k)
**I*6***	311.781	331.655	332.247	268.439	214.013
**I*8***	347.608	369.186	369.779	301.645	228.522
**I*10***	383.435	406.717	407.310	334.847	243.042
**I*12***	419.262	444.249	444.842	368.056	257.542

**Table 3:** Total Energy, E_HOMO_, E_LUMO_, ∆E, dipole moment, ionization energy, electron affinity and polarizability calculated for the present series, **I*n***.

Comp.	Total Energy (Hartree)	E_HOMO_ (ev)	E_luMO_ (ev)	∆E (ev)	Dipole Moment (Debye)	IE (ev)	EA (ev)	Polarizability Bohr^3^
**I*6***	−1500.642	−6.035	−2.062	3.973	6.2270	6.035	2.062	374.73
**I*8***	−1579.207	−6.034	−2.062	3.972	6.2646	6.034	2.062	398.87
**I*10***	−1657.771	−6.034	−2.061	3.973	6.2850	6.034	2.061	422.75
**I*12***	−1736.335	−6.033	−2.061	3.972	6.2981	6.033	2.061	446.49

Abbreviations; E_HOMO_; energy of the highest occupied molecular orbital, E_LUMO_; energy of the lowest unoccupied molecular orbital and ∆E = E_LUMO_−E_HOMO_; orbital energy gap.

**Table 4:** Summary of optical parameters evaluated from absorption and fluorescence spectroscopy.

Sample	Absorption			λ_emission_ (nm)	Life Time τ_avg_ (ns)
	λ_max_ (nm)	λ_Shoulder_ (nm)	E_g_ (eV)		
**I*6***	293	361	2.97	462	107
**I*8***	292	365	2.93	467	133
**I*10***	294	383	2.79	473	150
**I*12***	293	392	2.71	480	178

## Data Availability

The data presented in this study are available on request from the corresponding author.

## Supplementary Materials

The following are available online at www.mdpi.com/article/10.3390/polym14030456/s1, the synthetic and characterization details of investigated compounds.
