# Supplementary material for: New Self-Organizing Optical Materials and Induced Polymorphic Phases of Their Mixtures Targeted for Energy Investigations
Source: Polymers (Basel). 2022 Jan 23;14(3):456. doi: 10.3390/polym14030456 (PMC8838461; doi:10.3390/polym14030456)
Supplement: Supplementary file 1 [file polymers-14-00456-s001.zip › polymers-1560460-supplementary.pdf]

Supplementary Data

New optical materials for energy investigations and induced polymorphic phases of their mixtures

Salhah H. Alrefaee [1], Hoda A. Ahmed [1,2,*], Mohd Taukeer Khan [3], Khulood A. Al-Ola [4], Hanaa AL-Refai [1] and Mohamed A. El-Atawy [1,5]

[1] Department of Chemistry, College of Science, Taibah University, Yanbu 30799, Saudi Arabia; srfaay@taibahu.edu.sa (S.H.A.); hhjuhani@taibahu.edu.sa (H.A.-R.); Maatawy@taibahu.edu.sa (M.A.E.-A.)
[2] Department of Chemistry, Faculty of Science, Cairo University, Cairo 12613, Egypt
[3] Department of Physics, Faculty of Science, Islamic University of Madinah, Al-Madinah Al-Munawwarah 42351, Saudi Arabia; khanmtk@iu.edu.sa
[4] Chemistry Department, College of Sciences, Madina Monawara, Taibah University, Al-Madina 30002, Saudi Arabia; kabualola@taibahu.edu.sa
[5] Chemistry Department, Faculty of Science, Alexandria University, P.O. Box 426 Ibrahemia, Alexandria 21321, Egypt
* Correspondence: ahoda@sci.cu.edu.eg

1. Materials

4-Hexyloxylbenzoic acid, 4-octoyloxybenzoic acid, 4-decyloxybenzoic acid, 4-dodecyloxybenzoic acid, vanillin, and 4-floroaniline were purchased from Sigma Aldrich (Germany). Dichloromethane, *N,N'*- dicyclohexylcarbodiimide (DCCD), ethanol and 4-dimethylaminopyridine (DMAP) were purchased from Aldrich (Wisconsin, USA).

2. Synthesis

2.1. *Synthesis of 4-(((4-fluorophenyl)imino)methyl)-2-methoxyphenol*

Equimolar amount of vanillin (500 mg, 3.28 mmol) and 4-floroaniline (365 mg, 3.28 mmol) in ethanol (10 ml) were refluxed for 90 minutes. The reaction mixture was allowed to cool, and the precipitated product was filtered. The obtained solid was recrystallized from ethanol. The melting points were determined for the prepared imines accordance to the literature [30,31].

2.2. *Synthesis 4-(((4-fluorophenyl)imino)methyl)-2-methoxyphenyl 4-alkoxybenzoate,* **I*n***

Molar equivalents of 4-(((4-fluorophenyl)imino)methyl)-2-methoxyphenol and 4-alkoxybenzoic acid (0.01 mol) were dissolved in dry methylene chloride (DCM) (20 ml). 0.02 molar of *N*, *N'*-dicyclohexylcarbodiimide (DCC) and trace amount of 4-

dimethylaminopyridine (DMAP) were added to the reaction mixture. The reaction was left under stirring at room temperature for 72 hrs. The separated byproduct, dicyclohexylurea (DCU), was filtered off. The filtrate was then evaporated and the obtained product was recrystallized from ethanol.

3. Characterization

Perkin-Elmer B25 (Perkin-Elmer, Inc., Shelton, CT USA) spectrophotometer was used for infrared spectra measurements. Varian EM 350L 500 MHz spectrometer (Oxford, UK) was used for recording ^{1}HNMR spectra with tetramethyl silane as internal standard in $CDCl_3$; the chemical shift values recorded as δ (ppm units). Thermo Scientific Flash 2000 CHS/O Elemental Analyzer, Milan, Italy was used for Elemental analyses. TA Instruments Co. (Q20 Differential Scanning Calorimeter, DSC; USA) was used for recording phase transitions. DSC calibration was carried using lead and indium melting temperature and enthalpy. Samples of 2–3 mg were used in aluminum pans for DSC investigation. The heating rate was 10°C/min in nitrogen gas as an inert atmosphere (30 ml/min). All transitions measured for the second heating scan. Transition temperatures for the prepared compounds were checked and phases identified by Polarized optical microscope (POM, Wild, Germany) attached with Mettler FP82HT hot stage. Absorption spectra of prepared films were recorded through Agilent Cary 5000 UV-Vis-NIR spectrophotometer. The steady state emission and time resolved decay spectra were recorded through Horiba delta flex TCSPC lifetime fluorometer by exciting the sample from Delta-diode 320 nm with peak width ±10 nm.
